# Cytotoxic Effect of a β1,4-Galactosyltransferase Inhibitor in Hepatic Carcinoma Cells

**DOI:** 10.3390/cells15030251

**Published:** 2026-01-28

**Authors:** Zhe Dai, Ming Sun, Lihang Chen, Xueqi Fu, Wenfu Yan, Yin Gao, Inka Brockhausen

**Affiliations:** 1 Key Laboratory for Molecular Enzymology and Engineering of Ministry of Education, School of Life Sciences, Jilin University, Changchun 130012, China; 12231346@mail.sustech.edu.cn (Z.D.); sunming19@mails.jlu.edu.cn (M.S.); fxq@jlu.edu.cn (X.F.); 2 Joint Laboratory of Guangdong-Hong Kong Universities for Vascular Homeostasis and Diseases, Department of Pharmacology, School of Medicine, Southern University of Science and Technology, Shenzhen 518055, China; 3 SUSTech Homeostatic Medicine Institute, School of Medicine, Southern University of Science and Technology, Shenzhen 518055, China; 4 SKL of Marine Food Processing & Safety Control, National Engineering Research Center of Seafood, Collaborative Innovation Center of Seafood Deep Processing, School of Food Science and Technology, Dalian Polytechnic University, Dalian 116034, China; lhchen@xy.dlpu.edu.cn; 5 State Key Laboratory of Inorganic Synthesis and Preparative Chemistry, College of Chemistry, Jilin University, Changchun 130012, China; yanw@jlu.edu.cn; 6 Department of Biomedical and Molecular Sciences, Queen’s University, Kingston, ON K7L 3N6, Canada

**Keywords:** β1,4-galactosyltransferase 1 inhibitor, anticancer agents, cytotoxicity, cell cycle and apoptosis

## Abstract

**Highlights:**

**What are the main findings?**

- The β4GalT1 inhibitor **612** selectively suppresses proliferation of carcinoma cells with high B4GALTs expression.
- **612** suppresses hepatocellular carcinoma cells migration and invasion, induces ER and Golgi stress, triggers G2/M cell cycle arrest, and activates both intrinsic and extrinsic apoptosis pathways.

**What are the implications of the main findings?**

- Targeting β4GalT family-mediated glycosylation represents a promising therapeutic strategy for hepatocellular carcinoma with elevated expression of β4GalT family members.
- **612** shows potential as both a selective anti-cancer agent and an adjuvant to enhance apoptosis sensitivity in glycosylation-driven malignancies.

**Abstract:**

The incidence and mortality of hepatocellular carcinoma (HCC) are increasing worldwide, underscoring the need for novel therapeutic strategies. Synthetic 2-naphthyl 2-butanamido-2-deoxy-1-thio-β-d-glucopyranoside (**612**) is a selective inhibitor of β1,4-galactosyltransferase 1 (β4GalT1). In this study, we investigated the cytotoxic effects of **612** across multiple cancer cell lines, with a focus on HCC, and explored the underlying mechanisms. We demonstrate that **612** preferentially exhibits cytotoxicity toward cancer cells with elevated expression of β4GalT family members, while human umbilical vein endothelial cells and immortalized human embryonic kidney epithelial cells are comparatively less sensitive. Treatment with **612** suppresses cancer cell migration and invasion and induces pronounced endoplasmic reticulum and Golgi stress, accompanied by G2/M cell cycle arrest. Furthermore, **612** activates apoptosis through ER stress–associated pathways by downregulating the anti-apoptotic protein Bcl-2 and upregulating pro-apoptotic proteins Bax and Bak, along with activation of caspase-3, -8, and -9. Collectively, these findings identify **612** as a promising anti-cancer candidate targeting β4GalTs-overexpressing HCC cells and warrant further therapeutic development.

## 1. Introduction

Cancer is a life-threatening disease, and the discovery of new anticancer drugs with novel mechanisms of action and fewer side effects remains urgently needed. Cancer cells are known to exhibit aberrant glycosylation which plays a critical role in cancer progression, proliferation, invasion, angiogenesis and metastasis [1,2]. Glycosyltransferases (GTs) are responsible for the biosynthesis of complex glycoconjugates and are involved in the regulation of cell growth and cell adhesion [3,4]. Several GTs have been identified to be abnormally expressed in cancer and metastases, leading to the modification of glycosylation. These GTs are therefore potential targets for the development of cancer treatments [5,6].

The endoplasmic reticulum (ER) controls the synthesis, processing, packaging and transport of proteins in eukaryotic cells [7,8]. Changes in the tumor microenvironment such as hypoxia, aerobic glycolysis, presence of reactive oxygen species (ROS) or drug stimulation can lead to ER stress [9]. In response, misfolded proteins accumulate in the lumen of the ER leading to the unfolded protein response (UPR) [10]. UPR can re-establish homeostasis of the ER, but continuous ER stress can trigger cell apoptosis. Therefore, inducing ER stress and promoting the pro-apoptotic response may become a new strategy for treating cancer [11].

The β1,4-galactosyltransferases (β4GalTs) are involved in the synthesis of complex *N*- and *O*-linked *N*-acetyllactosamine chains of glycoproteins and lacto series of glycolipids. These *N*-acetyllactosamine structures form scaffolds for the attachment of Lewis epitopes that play crucial roles in cell adhesion of cancer and immune cells. Inhibition of *N*-acetyllactosamine biosynthesis can therefore affect the functions of glycoconjugates and lead to phenotypic changes of cancer cells [12,13,14]. Recent research indicated that *B4GALT1* and *B4GALT3* genes were up-regulated in liver tumor tissues of mice [15], while *B4GALT2*, *3*, *4*, *6* and *7* genes were up-regulated in human liver tumor tissues [16]. Human leukemia cells that highly express *B4GALT1* and *B4GALT5* exhibited multi-drug resistance, and knockdown of *B4GALT1* resulted in increased sensitivity to chemotherapeutic drugs [17,18,19].

Further modifications of the *N*-acetyllactosamine chains by fucosylation [20] and sialylation [21] generate adhesive epitopes, such as sialyl-Lewis x (SLe^x^) that can interact with selectins and have been correlated with cancer metastasis and poor survival rates in cancer patients [12,13,14]. The inhibition of SLe^x^ expression on cancer cell surfaces blocked selectin-mediated cell adhesion and reduced the metastatic potential of cancer cells [22,23].

GT inhibitors that modify cancer cell glycosylation have emerged as a promising family of anticancer chemotherapeutics [24]. Disaccharide substrate analogs that act as inhibitors of β4GalT1 provided an inhibitory effect on selectin-mediated tumor metastasis [25]. However, there is lack of information on the specificity and mechanisms of these inhibitors for other GTs. Glycoconjugates based on 8-hydroxyquinoline that inhibit β4GalT1 exhibited anti-cancer properties in cervical, breast, brain, pancreatic and colon cancer cell lines [26]. Analogs of GalNAcα-benzyl and tunicamycin inhibit *O*- and *N*-glycosylation, respectively, and induced apoptosis and blocked the proliferation of cancer cell lines [27,28,29]. Thus, the inhibition of glycosylation has been shown to prevent cancer cell metastasis [30,31] and to induce apoptosis in several types of cancer cells [28,32,33]. These collective findings strongly suggest that inhibitors of *N*- and *O*-glycosylation may represent potential anti-cancer agents.

Hepatocellular carcinoma (HCC) is a common primary liver malignancy. Despite intensive efforts to develop new therapeutic strategies, the incidence and mortality of HCC continue to rise worldwide, and systemic therapy remains a major option, particularly for patients with metastatic disease [34]. Aberrant glycosylation has been increasingly recognized as a hallmark of HCC progression, and several members of the β1,4-galactosyltransferase (β4GalT) family have been reported to be upregulated in liver tumors and to contribute to malignant phenotypes [15]. Although β4GalT-mediated glycosylation has been implicated in cancer progression, it remains unclear whether the selective vulnerability of hepatocellular carcinoma cells reflects a broader dependency on β4GalT-associated glycosylation programs. In particular, how disruption of β4GalT-related glycosylation influences cellular stress responses and survival in HCC cells has not been fully defined.

In previous studies, we developed and characterized 2-naphthyl 2-butanamido-2-deoxy-1-thio-β-D-glucopyranoside (**612**) as a specific inhibitor against β4GalT1 and this specificity refers to inhibition of β4GalT1 and does not imply inhibition of related galactosyltransferases such as β3GalT5 [35]. Building on this work, the present study focuses on the anti-cancer activity of **612** in HCC cells and aims to elucidate the underlying cellular mechanisms. We hypothesized that HCC cells with elevated β4GalT-associated glycosylation would exhibit increased sensitivity to treatment with compound **612**, leading to glycosylation alterations. We have also evaluated the cytotoxic selectivity of **612** across multiple cancer and non-malignant cell lines, examined its effects on cell-surface glycosylation and associated migratory and invasive behaviors, and assessed whether treatment with **612** induces ER and Golgi stress, G2/M cell-cycle arrest, and apoptotic signaling in HCC cells. By integrating in vitro functional assays with bioinformatic analyses of human HCC datasets, this study highlights the anti-cancer potential of **612** as a glycosylation-targeting compound and provides a basis for its further investigation.

## 2. Materials and Methods

### 2.1. Reagents

Phosphate buffered saline (PBS) and Trizol reagent were obtained from Monad (Suzhou, China). Trypsin-EDTA solution, DAPI and Bovine Serum Albumin (BSA) were from Solarbio (Beijing, China). Antibodies against Bcl-2, Bax, caspase-3, caspase-9, caspase-8, ATF6, IRE1α, PERK, CHOP, Bak, GRP78, ARF4, P-JNK, GAPDH, Tubulin and β-actin were obtained from Affinity (Cincinnati, OH, USA). Rabbit polyclonal anti-Fas antibody and FITC-conjugated Fas Antibody, as well as unconjugated anti-Lewis a, anti-Lewis b, anti-Lewis y and anti-sialyl-Lewis x were obtained from Abcam (Beijing, China). Human E-selectin Fc and P-selectin were obtained from R&D systems (Minneapolis, MN, USA). Anti-β4GalT1 antibody was obtained from Santa Cruz Biotechnology (Dallas, TX, USA). The secondary antibodies were peroxidase-conjugated and obtained from Nachuan (Changchun, China). FITC-conjugated secondary antibodies were obtained from Abxin (Shanghai, China). Matrigel was obtained from Corning (Lowell, MA, USA). Phosphatase-conjugated avidin and nitrophenyl-phosphate were from Sigma-Aldrich (Shanghai, China). Crystal violet was from Guangfu (Tianjin, China). Hieff^®^ qPCR SYBR Green Master Mix (Yeasen Biotech Co., Shanghai, China) and 5× All-In-One RT MasterMix Kit was from ABM (Nanjing, China). Live cell counting kit-8 (CCK-8), RIPA lysis buffer, Super ECL detection reagent, cell apoptosis detection kit (TransDetect Annexin V-FITC/PI) were from Yeasen Biotech Co (Shanghai, China). Cell Cycle and Apoptosis Analysis Kit was from Beyotime (Shanghai, China). CellEvent^TM^ caspase-3/7 Green Detection Reagent was obtained from Thermo Fisher Scientific (Waltham, MA, USA). Anti-Fas antibody CH-11 was from Millipore (Burlington, MA, USA). 4% paraformaldehyde was obtained from Biosharp (Hefei, China).

### 2.2. Synthesis and Preparation of ***612*** for Cell Culture Experiments

The β4GalT inhibitor **612** was synthesized as described previously and the structure was confirmed as 2-naphthyl 2-butanamido-2-deoxy-1-thio-β-d-glucopyranoside characterized via ^1^H NMR and LC-ESI-MS analysis [35,36] as follows: ^1^H NMR (600 MHz, 6 mg dissolved in 500 μL DMSO-*d*_6_): δ 0.89 (t, 3 H, *J* 7.4 Hz, CH_3_CH_2_CH_2_), 1.55 (m, 2 H, *J* 7.3 Hz, CH_3_CH_2_CH_2_), 2.07 (t, 2 H, *J* 7.2 Hz, CH_2_CO), 3.14–3.75 (other Hs on carbohydrate ring), 4.88 (d, 1 H, *J* 10.3 Hz, anomeric H on carbohydrate ring), 7.46–7.95 (7 H, on the naphthyl ring). ^13^C NMR (150.9 MHz, DMSO-*d*_6_): δ 14.15 (CH_3_CH_2_CH_2_), 19.16 (CH_3_CH_2_CH_2_), 38.31 (CH_2_CO), 54.72–81.86 (other Cs on carbohydrate ring), 87.01 (anomeric C on carbohydrate ring), 126.23, 127.02, 127.38, 127.66 (2 C), 128.03, 128.57, 131.82, 133.81, and 133.93 (Cs on the naphthyl ring), 172.46 (NCOCH_3_). HRMS (ES): calculated for C_20_H_26_NO_5_S ([M + H]^+^) was 392.1532. Actual mass found was 392.1439.

Inhibitor **612** was dissolved in dimethyl sulfoxide (DMSO) (Dingguo, Beijing, China) with a concentration of 100 mg/mL as a stock solution and stored at −20 °C. The stock solution was diluted with growth medium to obtain various final concentrations of **612** for the cell culture experiments. In all experiments, control groups were treated with the same final concentration of DMSO as the corresponding **612**-treated groups, which was 0.1% (*v/v*).

### 2.3. Cell Cultures

HUVEC, HEK293A, HCC cells (SMMC-7721 and HepG2), human breast cancer cells (MCF-7 and MDA-MB-231), human lung cancer cells (A549), and human colorectal adenocarcinoma cells (SW480) were obtained from the Cell Bank of the Committee on Type Culture Collection of Chinese Academy of Sciences (Shanghai, China). Complete cell growth medium RPMI 1640 and DMEM were obtained from Gibco, Invitrogen (Paisley, UK), fetal bovine serum (FBS) and penicillin/streptomycin were from Thermo Fisher Scientific (Shanghai, China). HUVEC were cultured in RPMI 1640 medium. MCF-7, MDA-MB-231, HEK293A, A549, SMMC-7721, SW480 and HepG2 were cultured in DMEM medium. Cell cultures were supplemented with 10% FBS and 1% penicillin/streptomycin (Solarbio Beijing, China) and incubated at 37 °C in a humidified atmosphere containing 5% CO_2_.

### 2.4. Cell Viability Assays

To measure cell viability as an indicator of cytotoxicity, cells in logarithmic growth phase (1 × 10^4^) were seeded into wells of a 96-well plate and grown in complete growth medium for 24 h in a CO_2_ incubator and evaluated using the CCK-8, which measures metabolic activity as an indicator of viable cell number. This assay was employed to quantitatively evaluate the cytotoxic effects of compound **612** and to determine appropriate working concentrations for subsequent functional assays. Experimental groups were treated with various concentrations of **612** (10, 20, 50, 100, 200, 500 μg/mL, which are 25.5, 51, 127, 255, 510, 1275 μM respectively) for 24 h in the absence of FBS (serum-free). Groups that lacked **612** were used as controls. Cells were then incubated with 10 µL of CCK-8 solution at 37 °C for 1–4 h. The optical density (OD) was measured at 450 nm, and each measurement was repeated 6 times. The working concentrations of **612** (up to 500 µg/mL) were selected based on preliminary titration experiments and the known characteristics of carbohydrate-based glycosyltransferase inhibitors, which often require high extracellular concentrations to overcome limited membrane permeability and achieve meaningful intracellular effects in vitro [37]. Cells were cultured under serum-free conditions to minimize potential interference from serum-derived glycosyltransferase activities, such as β4GalT1, which could confound the assessment of glycosylation-dependent effects of compound **612**. All treatment and control groups were maintained under identical serum-free conditions to ensure valid comparisons.

### 2.5. Migration and Invasion Assays

For the scratched wound healing assays, SMMC-7721 and HepG2 cells were grown with complete growth medium in 6-well plates until reaching 90% confluence; then a scratch was made with a pipette tip through the monolayer in the middle of each well. Cells were washed and switched to serum-free medium containing **612** at 12.5 μg/mL, 50 μg/mL or 100 μg/mL. Groups that lacked **612** were used as controls. Images were taken using a microscope (LIOO, Beijing, China) at 0, 24, and 48 h of the treatment. Wound closure was quantified as relative closure normalized to the initial scratch area, and all comparisons were performed using this normalized metric. The change in scratch width was measured by image J.

For migration assays, cells were seeded into transwell inserts with an 8.0 μm pore size. Cells were starved for 24 h prior to seeding in the inserts of a 24-well plate (Corning, Lowell, MA, USA). Serum-free medium (200 μL, 1.5 × 10^4^ cells) containing 50 μg/mL **612** was added to the insert and 600 μL 10% FBS serum medium were added to the lower chamber of the 24-well plate as a chemoattractant. After 24 h incubation, non-migrating cells were removed by wiping the upper side of the insert with a cotton swab. Migrated cells on the bottom side of the insert were quantified using a microscope and migrated cells were counted.

Transwell-invasion assays were performed via a similar method to the migration assays, except that the cells were seeded on top of Matrigel coating. Matrigel was thawed and liquefied on ice, then 50–60 μL were added to a 24-well transwell insert and solidified in a 37 °C incubator for 30 min to form a thin gel layer. Cells were starved for 24 h prior to seeding on top of the Matrigel.

### 2.6. Lectin Staining Assays

Lectin staining assays were performed to assess cell surface glycosylation via a previously reported method [38]. Briefly, cells were treated with 50 μg/mL **612** for 48 h. Lectin staining was performed on cells that were fixed in 96-well microtiter plates and incubated with biotinylated lectins (Vector Labs, CA, USA): Ricinus communis agglutinin I (RCA I) that recognizes Gal, Maackia amurensis Lectin II (MAL II) that binds Neu5Acα2-3, Sambucus nigra lectin (SNA) that binds Neu5Acα2-6, Concanavalin A (ConA) that recognizes mannose, Datura stramonium (DSL) and Wheat germ agglutinin (WGA) that binds GlcNAc, Maackia amurensis lectin I (MAL I) that recognizes Galβ1-4GlcNAc and Phaseolus vulgaris Leukoagglutinin (PHA-L) that recognizes Galβ1-4GlcNAcβ1-6(GlcNAcβ1-2Manα1-3)Manα1-3. This was followed by incubation with alkaline phosphatase-conjugated avidin and nitrophenyl-phosphate reaction substrate. The absorbance was measured with a microplate reader at 405 nm.

### 2.7. ELISA Assays

In order to assess adherence of cells to selectins, E-selectin Fc or P-selectin Fc (0.5 μg/mL) were coated on an ELISA plate at 4 °C overnight. 20,000 **612** pre-treated cells and control cells were added to the corresponding wells and incubated for 2 h at 37 °C. Wells were washed with PBS gently, then fixed with 10% formaldehyde and air-dried. The adhesive cells were stained with crystal violet (0.05%). Images were taken using a microscope (LIOO, Beijing, China).

### 2.8. Analysis of Cell Surface Structures

Cell surface carbohydrate epitopes were examined using fluorescently labeled antibodies. Cells were seeded into coverslips (NEST, Wuxi, China) in 24-well plates (1 × 10^5^) and cultured for 24 h, followed by replacement of the growth medium with serum-free medium containing **612**, and without **612** as controls. The medium was withdrawn after another 24 h incubation, and cells were fixed with 4% paraformaldehyde. 5% BSA was then added for blocking, followed by the addition of 300× diluted primary antibody and cells were incubated overnight at 4 °C. After gently washing, the secondary antibody (300×) was added to the wells and incubated at room temperature for 1 h, and then counter-stained with diluted 4′,6-diamidino-2-phenylindole (DAPI) and analyzed with a confocal laser scanning microscope LSM 710 (Zeiss, Oberkochen, Germany).

### 2.9. Measurement of Apoptosis and Cell Cycle Analysis

The number of cells undergoing apoptosis were measured by flow cytometry (BD Biosciences, Franklin Lakes, NJ, USA). Cells collected from logarithmic growth phase (3 × 10^5^) were seeded into a 6-well plate and pre-incubated for 24 h, then switched to serum-free medium prior to the addition of **612**. After another 24 h incubation with **612**, cells were washed with pre-cooled PBS and binding buffer was employed to re-suspend cells. Annexin V-FITC and PI staining solution were added prior to loading cells into a flow cytometer according to the manufacturer’s instructions of the apoptosis kits. For the cell cycle analysis, the pre-treated cells were washed with pre-cooled PBS, followed by a fixation with 75% cold ethanol for 24 h at −20 °C. According to the instructions of the cell cycle detection kit, PI staining solution was added for flow cytometry detection. The fluorescence spectra were recorded after the staining steps and the total number of cells detected was 20,000 each time.

### 2.10. Western Blot Analysis

Protein expression was examined by Western blots (WB). Cells were incubated with 100 μg/mL of **612** for 6, 12 and 24 h before total proteins were extracted and measured using a bicinchoninic acid (BCA) protein concentration assay kit (Biyuntian, Beijing, China). Proteins were separated via SDS-PAGE for 90 min and transferred to polyvinylidene fluoride (PVDF) membranes (Invitrogen), with β-actin, Tubulin or GAPDH serving as loading controls. The membranes were incubated with primary antibodies (1000× dilution) at 4 °C overnight. After washing with Tris-buffered saline-tween (TBST) (0.05% Tween 20) the secondary antibody (10,000× dilution) was added to the membranes and incubated for 2 h. The enhanced chemiluminescence (ECL) developing solution (Yeasen Biotech Co., Shanghai, China) was added to the PVDF membrane, and the results were recorded by Gel imaging system (Tanon, Shanghai, China).

### 2.11. RT-qPCR

To measure mRNA expression levels, cells were seeded into a six-well plate and cultured for 24 h. The medium was removed and 1 mL of Monzol was added to extract RNA from cells. The RNA concentration was measured with Nanodrop (ThermoFisher Scientific, Waltham, MA, USA). The 5× All-In-One RT MasterMix Kit (abm, Nanjing, China) was used to reverse transcribe RNA into DNA. The qPCR reaction was performed with Hieff^®^ qPCR SYBR Green Master Mix (Yeasen Biotech Co., Shanghai, China). ABM7500 qPCR instrument was used for real-time quantification. Primer sequences are listed in Table S1 in the Supplementary Information.

### 2.12. TCGA and GTEx Data Acquisition and Preprocessing

RNA-sequencing data of hepatocellular carcinoma (HCC) and normal liver tissues were obtained from The Cancer Genome Atlas (TCGA) and the Genotype-Tissue Expression (GTEx) project. Specifically, 371 HCC samples from TCGA and 160 normal liver samples from TCGA and GTEx were included in the analysis. To ensure cross-cohort consistency and minimize computational batch effects, we utilized the UCSC Toil RNA-seq recompute dataset, which applies a unified processing pipeline across TCGA and GTEx cohorts. Gene expression values were analyzed at the transcript per million (TPM) level. Batch effects between TCGA and GTEx datasets were corrected using the sva R package (https://bioconductor.org/packages/release/bioc/html/sva.html) (accessed on 25 June 2025).

### 2.13. Single-Sample Gene Set Enrichment Analysis (ssGSEA)

To quantify the pathway activity of glycosyltransferase-related signatures, single-sample gene set enrichment analysis (ssGSEA) was performed using the GSVA R package (v1.46.0). The B4GALT score was calculated based on the expression of B4GALT1-7. The SLe^x^ score was derived from a curated gene set including B3GNT2-8, GCNT2, GCNT3, ST3GAL1-6, and FUT3-7, FUT9, representing key enzymes involved in the biosynthesis of SLe^x^. Regarding multiple-testing correction, this analysis was hypothesis-driven and specifically designed to test the predefined biological relationship between B4GALT expression and SLe^x^ biosynthesis. Therefore, a standard Spearman correlation test was applied.

### 2.14. Statistical Analyses

GraphPad Prism 7 was employed in this study for the statistical analysis of CCK-8, flow cytometry and WB and data were presented as mean ± standard deviation (SD). In our data * *p* < 0.05, ** *p* < 0.01, *** *p* < 0.001 (using Student’s *t*-test and one-way ANOVA) were considered statistically significant. The statistical analysis in the bioinformatics analysis was conducted using R (v4.2.2).

## 3. Results

### 3.1. Cytotoxicity of ***612***

**Figure 1:**
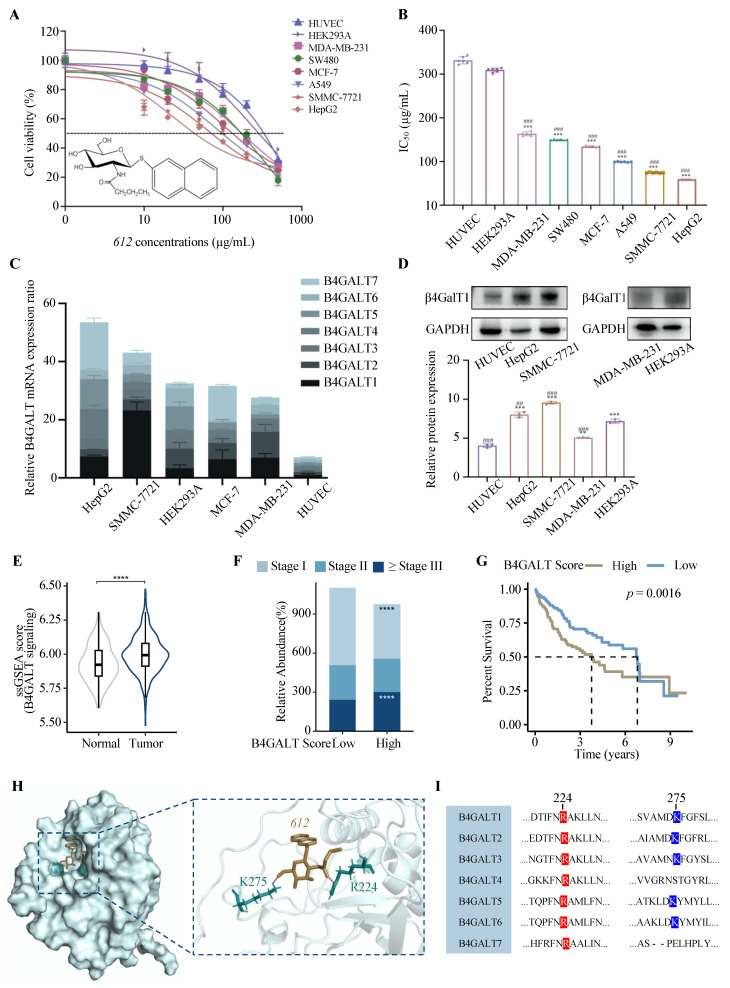
Cytotoxicity of **612**. (**A**) Cell viability (%) of HUVEC and a group of cancer cells after **612** treatments was evaluated by CCK-8 assay at 24 h. Data shown are the mean ± SD of six separate experiments. The horizontal dashed line indicates 50% cell viability, which is used to determine the IC_50_ values. (**B**) The IC_50_ of **612** against various cell lines. Data shown are the mean ± SD of six separate experiments. *** *p* < 0.001, cancer cells compared to HUVEC cells. ^###^ *p* < 0.001, cancer cells compared to HEK293A cells. (**C**) The expression of B4GALTs mRNA in HepG2, SMMC-7721, HEK293A, MCF-7, MDA-MB-231 and HUVEC by RT-qPCR. (**D**) The expression levels of β4GalT1 proteins in HUVEC, HepG2, SMMC-7721, MDA-MB-231 and HEK293A cells as determined via western blot analysis. Data shown are the mean ± SD of six separate experiments. ** *p* < 0.01, *** *p* < 0.001, cells compared to HUVEC cells. ^##^ *p* < 0.01, ^###^ *p* < 0.001, cells compared to HEK293A cells. (**E**) The ssGSEA score of B4GALTs signaling in TCGA and GTEx datasets. **** *p* < 0.0001. (**F**) Stage composition of high- vs. low-B4GALTs score groups, classified using the cohort mean as cutoff. **** *p* < 0.0001. (**G**) Survival analysis of high- vs. low-B4GALTs score groups in HCC. The dashed lines indicate the median survival time (50% survival probability) for each group. (**H**) Docking of a **612** molecule into the crystal structure of β4GalT1 (PDB: 6FWT). (**I**) Sequence alignment of interaction sites across human B4GALT family members. Conserved residues potentially involved in **612** binding are highlighted.

The cytotoxic effects of **612** against HUVEC, HEK293A and a group of cancer cell lines were evaluated via CCK-8 assays that provide accurate live cell counting (Figure 1A). The half-maximal inhibitory concentrations (IC_50_) of **612** in all cell lines calculated via GraphPad Prism 7 are shown in Table S2 and Figure 1B. The highest IC_50_ value was observed in HUVECs (331.6 ± 8.3 μg/mL, corresponding to 845.0 ± 21.2 μM), indicating the weakest cytotoxicity, followed by human embryonic kidney HEK293A cells (309.2 ± 4.7 μg/mL, 788.2 ± 12.0 μM). In contrast, all cancer cell lines exhibited lower IC_50_ values, ranging from 163.1 ± 4.6 μg/mL (415.6 ± 11.7 μM) to 59.2 ± 0.7 μg/mL (150.9 ± 1.8 μM). The lowest IC_50_ values, and thus the strongest cytotoxicity, were observed in the HCC cell lines SMMC-7721 (74.3 ± 0.9 μg/mL, 189.4 ± 2.3 μM) and HepG2 (59.2 ± 0.7 μg/mL, 150.9 ± 1.8 μM). Taken together, these findings suggest that **612** could act as a cytotoxic agent with higher efficacy against cancer cells derived from different tissues than against normal cells.

### 3.2. Expression of β4GalTs in Cancer and Normal Samples

In order to explore the relationship between cytotoxicity of β4GalT inhibitor **612** and enzyme expression, the gene expressions of B4GALT family and the protein expression levels of β4GalT1 were measured in HUVEC, HEK293A, MDA-MB-231, and the two HCC cell lines, HepG2 and SMMC-7721 (Figure 1C,D). The expression levels of *B4GALT 1* to *7* genes were relatively low in HUVEC and at higher levels in the hepatocarcinoma cells. This was supported by WB analysis that showed lower β4GalT1 protein levels in HUVEC cells (Figure 1D). Single-sample gene set enrichment analysis (ssGSEA) of TCGA and GTEx datasets revealed significantly elevated B4GALT family signaling in tumor tissues compared to normal samples (Figure 1E). Subjects with elevated B4GALT expressions exhibited significantly more advanced cancer stages (Figure 1F). Activation of B4GALT signal correlated with poor survival among HCC patients. Moreover, structural docking supported the **612**-binding capacity of β4GalT1 (−6.8 kcal/mol). Two conserved residues in β4GalTs (R224 and K275) formed direct contacts with **612** in β4GalT1 catalytic pocket (Figure 1H,I and Figure S1). These findings suggest that enhanced β4GalT activity is associated with tumor progression and support the potential of β4GalT inhibition **612** as a therapeutic strategy. However, while these analyses suggest potential engagement of β4GalT family-associated glycosylation programs, direct functional evidence distinguishing β4GalT1-specific inhibition from broader β4GalT family inhibition remains limited in the present study.

### 3.3. Inhibitor ***612*** Prevents Cancer Cell Migration and Invasion

**Figure 2:**
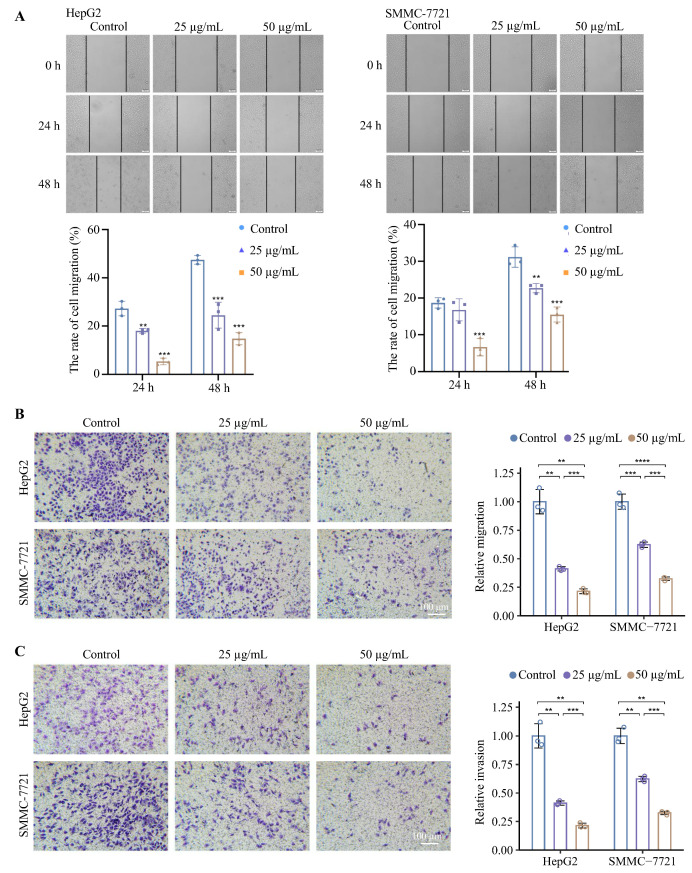
Anti-migration activity of **612**. The migration activity of HepG2 and SMMC-7721 was assessed with (**A**) Scratched wound healing assays (scale bar: 100 μm) and (**B**) Transwell assays. Data represent the mean ± SD of three independent experiments (** *p* < 0.01, *** *p* < 0.001, **** *p* < 0.0001, compared to the **612**-free control groups). (**C**) Transwell invasion assay. Data shown are the mean ± SD of three separate experiments (*** *p* < 0.001, compared to the **612**-free control groups).

Scratched wound healing and transwell assays were performed to evaluate the anti-migration activity of **612** in SMMC-7721 and HepG2 cell cultures. As shown in Figure 2A and Figure S2 the addition of **612** (12.5 μg/mL, 25 μg/mL and 50 μg/mL) resulted in slower wound healing when compared with the **612**-free controls. To further confirm this observation, transwell migration assays and Matrigel-coated transwell invasion assays were performed with the cells that were grown for 24 h with or without **612**. The results showed that treatment with **612** significantly reduced cell migration and decreased the ability of HCC cells to traverse the Matrigel-coated membrane (Figure 2B,C). As uncoated migration controls were not included in the initial invasion assays, these results reflect impaired ECM traversal capacity rather than a strictly normalized invasion index. In addition, it should be noted that, at higher concentrations, reduced cell viability and cellular stress responses induced by **612** may partially contribute to the observed inhibition of migration and invasion.

**Figure 3:**
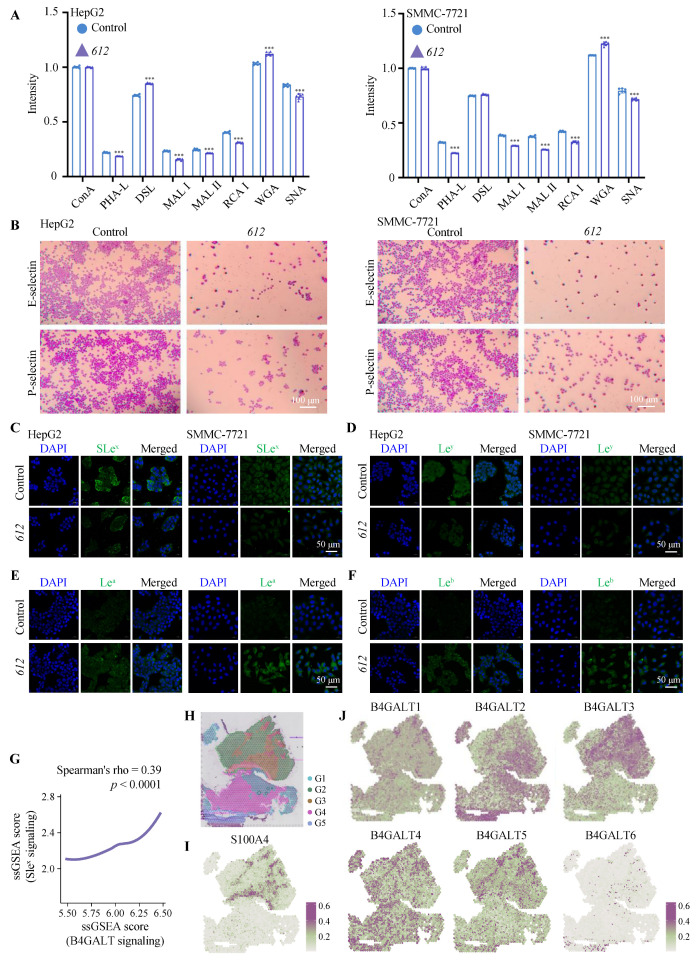
Cell surface glycosylation was modified by **612**. (**A**) Lectin-staining assays. Results are expressed as mean ± SD, *** *p* < 0.001. (**B**) Binding ability of cancer cells to E- and P-selectin. (**C**–**F**) The expression changes of SLe^x^, Le^y^, Le^a^ and Le^b^ on the cell surface by immunofluorescence. (**G**) Correlation analysis between B4GALTs scores and SLe^x^ levels. (**H**) Cluster labels of GSM6177612. (**I,J**) Standardized expression patterns of S100A4 and B4GALTs in a human HCC spatial transcriptomics dataset. The color gradient (gray to green to purple) indicates normalized relative expression levels of individual B4GALT family members in the spatial transcriptomics dataset, with purple representing higher expression. Values are scaled (0–0.6) for comparative visualization.

To verify that cell surface glycosylation was modified by **612**, lectin-staining assays were employed. As seen in Figure 3A, the number of Gal residues (recognized by PHA-L, MAL I, and RCA I lectins) and sialic acid residues (recognized by MAL II and SNA lectins) was reduced but the number of terminal GlcNAc residues (recognized by DSL and WGA lectins) was increased in **612** treated HCC cells. E- and P-selectin-coated plates were used to assess the binding ability of cancer cells to E- and P-selectins upon **612** treatments. As seen in Figure 3B, the number of cells that bind to E- and P-selectins was significantly reduced after **612** treatment (50 μg/mL), suggesting that the anti-migration activity of **612** could be due to the reduction of E- and P-selectin ligand, such as SLe^x^ expression on cancer cell surfaces. Consistently, immunofluorescence showed that the expressions of SLe^x^ and Le^y^ were decreased after the **612** treatments (Figure 3C,D), while the expressions of Le^a^ and Le^b^ were increased (Figure 3E,F). In evaluating the TCGA-HCC dataset, we found that the B4GALTs activity was significantly correlated with the score of SLe^x^ signaling (Figure 3G). We examined the expression patterns of B4GALTs by analyzing publicly available spatial transcriptomic datasets from HCC (Figure 3H, GSM6177612). In the spatial transcriptomic dataset, several B4GALT family members implicated in *N*-acetyllactosamine/SLe^x^ glycan biosynthesis showed higher transcript levels in S100A4^+^ regions compared with other tumor areas (Figure 3H,I). Among them, B4GALT4 and B4GALT5 displayed the most apparent enrichment in these regions (Figure 3J), whereas B4GALT6 did not show a similar pattern. Although this dataset does not involve compound **612** treatment, the spatial enrichment of selected B4GALT transcripts in S100A4^+^ regions is consistent with a potential role of β4GalT-associated glycosylation in invasive tumor compartments. This observation provides context for our in vitro findings that **612** perturbs cell-surface glycosylation and is associated with reduced migratory behavior.

### 3.4. Cell Cycle Arrest Induced by ***612***

**Figure 4:**
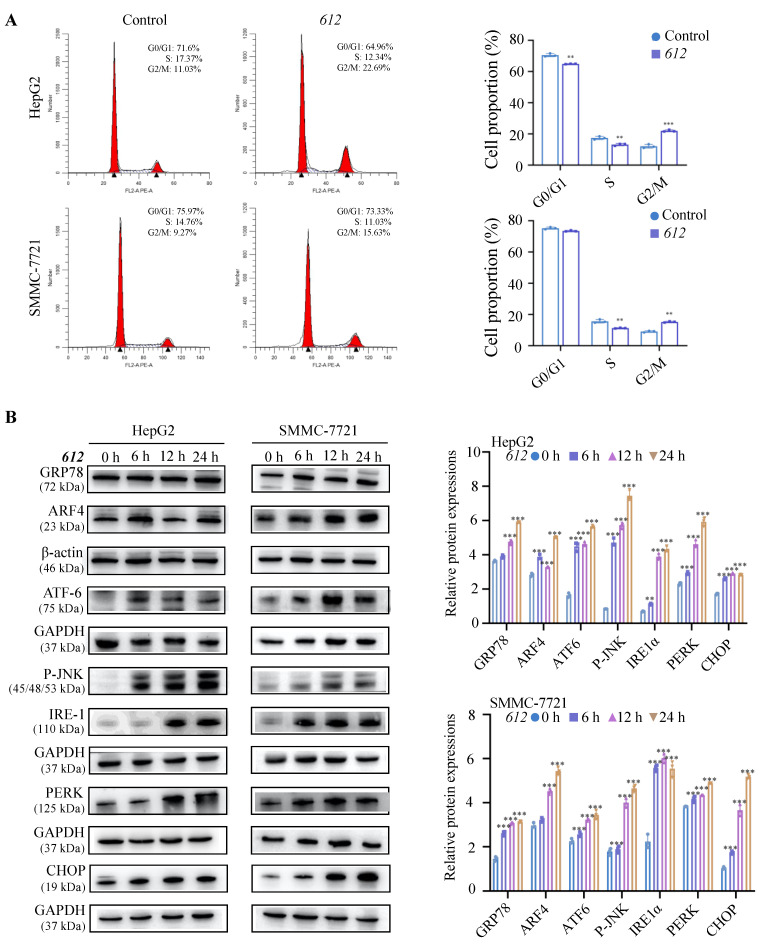
**612** induces cell cycle arrest and triggers ER/Golgi stress. (**A**) Analysis of changes in the HepG2 and SMMC-7721 cell cycles via flow cytometry after treatment with **612** (100 μg/mL) for 24 h. ** *p* < 0.01, *** *p* < 0.001, compared to the control groups. (**B**) The expression levels of ER/Golgi stress markers (GRP78, ARF4, ATF6, PJNK, IRE1α, PERK and CHOP) in SMMC-7721 and HepG2 cells after treatment with **612** (100 μg/mL) prior to examination by western blot analysis. ** *p* < 0.01, *** *p* < 0.001, compared to the control group.

In order to test if the anti-proliferation effect of **612** was due to cell cycle arrest, SMMC-7721 and HepG2 cell lines were incubated with serum-free growth medium containing 100 μg/mL **612** for 24 h, stained with propidium iodide (PI) and subjected to cell cycle analysis via flow cytometry. DNA in the cell stained by the fluorescent dye PI showed different fluorescence intensity at different periods of cell growth. As shown in Figure 4A, **612** significantly increased the percentage of HepG2 cells in the G2/M phase (*p* < 0.001) and decreased the percentage of cells in the G0/G1 and S phases (*p* < 0.01). This suggested that treatment with **612** led to cell cycle arrest at G2/M phase. Upon the **612** treatments, the distribution of SMMC-7721 cells in the G2/M phase also increased (*p* < 0.01), while the distribution in S phase decreased (*p* < 0.01). This suggests that **612** induced G2/M phase cell cycle arrest in both HCC cell cultures which is consistent with its cytotoxic effects.

### 3.5. ER and Golgi Stress Induced by ***612***

WB was used to determine the effects of **612** on the ER and Golgi stress in SMMC-7721 and HepG2 cells. Cells grown in the serum-free medium containing 100 μg/mL **612** were subjected to analysis of the expression levels of stress-related transcription factors over time. As seen in Figure 4B, the expression levels of ER stress-associated proteins increased with time, indicating that **612** induced ER stress in cancer cells associated with up-regulation of the expression of genes encoding GRP78 (*p* < 0.001), ATF6 (*p* < 0.001), P-JNK (*p* < 0.001), IRE1α (*p* < 0.01), PERK (*p* < 0.001) and CHOP (*p* < 0.001). Furthermore, Golgi stress-related protein ARF4 was also significantly increased after **612** treatment (*p* < 0.001).

### 3.6. Effect of ***612*** on Cancer Cell Apoptosis

**Figure 5:**
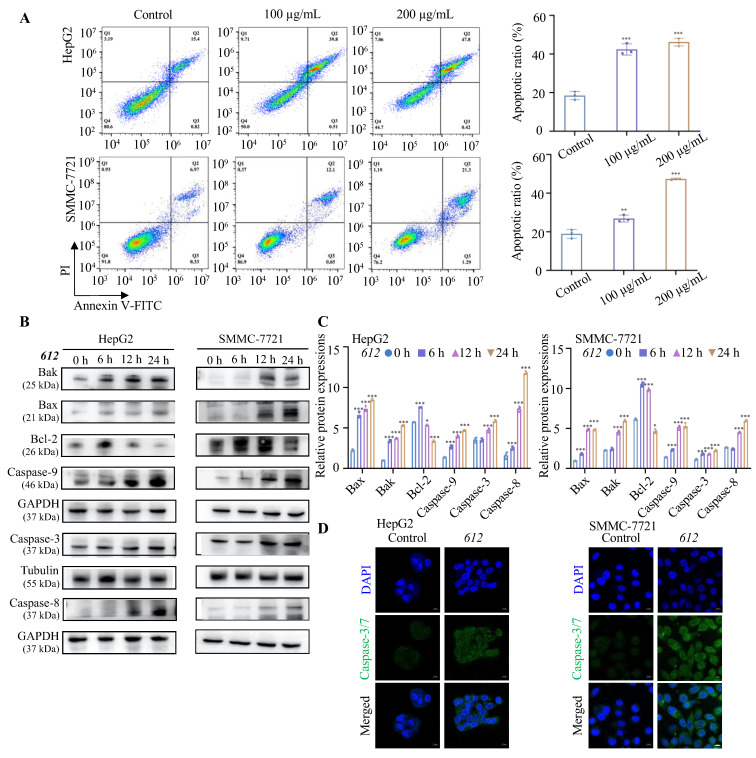
**612** induces intrinsic apoptosis in hepatocellular carcinoma cells. (**A**) The apoptosis level of HepG2 and SMMC-7721 after treatment with **612** (100 μg/mL and 200 μg/mL) determined after 24 h via flow cytometry. ** *p* < 0.01, *** *p* < 0.001. (**B**,**C**) The expression levels of apoptosis-related proteins (Bak, Bax, Bcl-2, Caspase-9, Caspase-3 and Caspase-8) in SMMC-7721 and HepG2 cells after treatment with **612** (100 μg/mL) prior to examination by western blot analysis. * *p* < 0.05, *** *p* < 0.001. (**D**) The fluorescence intensity of caspase-3/7 of HepG2 and SMMC-7721 treated with 100 μg/mL **612** by immunofluorescence assay. Scale bars, 10 μm.

To determine the level of apoptosis in HepG2 and SMMC-7721 cells after 24 h incubation with 100 or 200 μg/mL **612**, Annexin V-FITC and PI staining were used and analyzed by flow cytometry. As shown in Figure 5A, apoptosis of SMMC-7721 (*p <* 0.01) and HepG2 (*p* < 0.001) was significantly higher upon **612** treatments (100 μg/mL), and increased further upon 200 μg/mL **612**. HepG2 cells showed a higher apoptotic ratio indicating that they were more sensitive to **612**-induced apoptosis than SMMC-7721 cells.

To further unravel the apoptosis pathway triggered by **612**, the expression levels of apoptosis-related factors that mainly contribute to receptor-independent cell death were investigated via WB analysis. When cancer cells were treated with serum-free medium containing 100 μg/mL **612**, the expression levels of Bcl-2 family molecules changed in a time-dependent manner (Figure 5B,C). The expression of pro-apoptotic factor Bax and Bak increased in all cancer cells, whereas the anti-apoptotic factor Bcl-2 was suppressed. Furthermore, the expression levels of caspase-3, -8 and -9 increased with longer **612** incubation times. This analysis indicated that **612** stimulated the intrinsic apoptosis pathway in cancer cells through upregulation of the pro-apoptosis protein factors Bax (*p* < 0.001) and Bak (*p* < 0.001), downregulation of the anti-apoptotic factor Bcl-2 (*p* < 0.05), and the activation of apoptosis initiators caspase-9 (*p* < 0.001) and caspase-8 (*p* < 0.001), which eventually led to the activation of the effector caspase-3 (*p* < 0.001). In addition, the results of immunofluorescence experiments showed that the fluorescence intensity of caspase-3/7 of HepG2 and SMMC-7721 cells treated with **612** was higher compared to the untreated group (Figure 5D). The caspases were expressed outside of the nucleus. Consistent with prolonged exposure and robust stress induction, **612** treatments resulted predominantly in Annexin V^+^/PI^+^ cell populations, indicative of late-stage apoptotic events.

**Figure 6:**
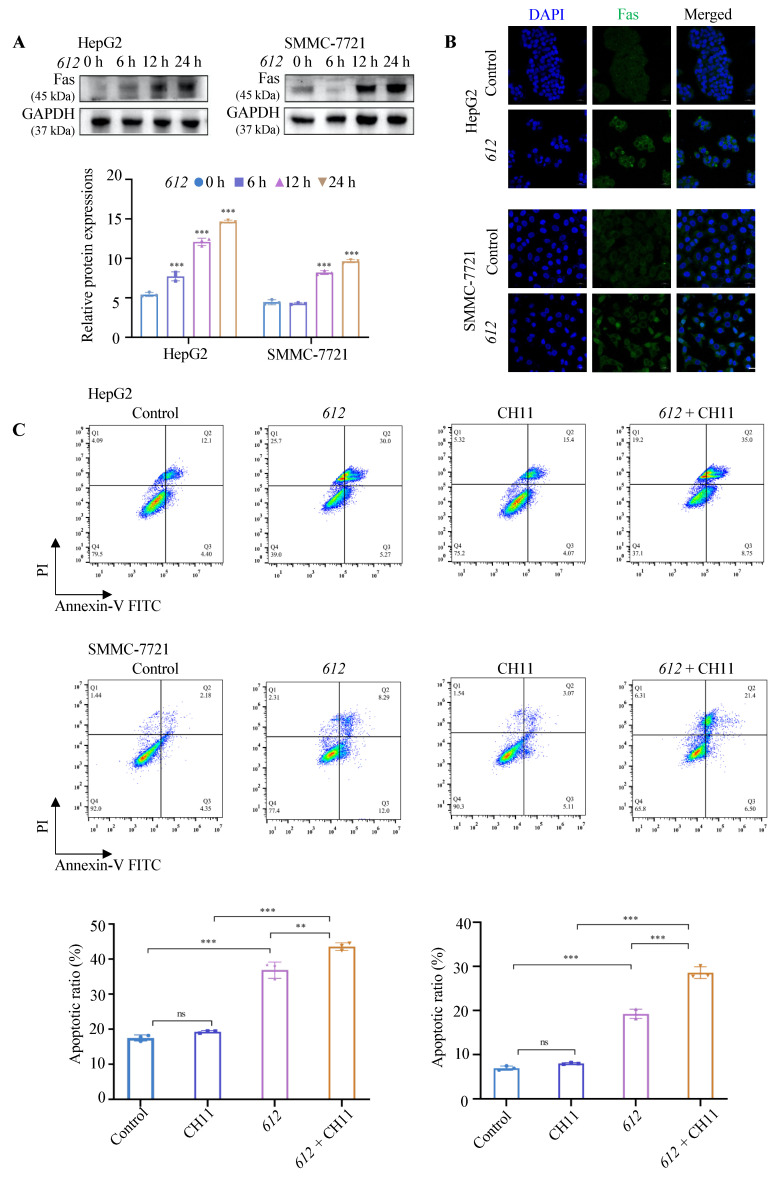
**612** enhances Fas-mediated extrinsic apoptosis in hepatocellular carcinoma cells. (**A**) Expression of Fas by Western blot analysis. *** *p* < 0.001, **612**-treated (100 μg/mL) groups compared with the control group. (**B**) The expression of Fas receptor on the surface of **612**-treated (100 μg/mL) HepG2 and SMMC-7721 by immunofluorescence. Scale bars 20 μm. (**C**) The apoptosis level of HepG2 and SMMC-7721 after treatment with 100 μg/mL **612**, followed by 20 ng/mL Fas ligand CH11 and double processing via flow cytometry. ** *p* < 0.01, *** *p* < 0.001, “ns” stands for “not significant”, compared with the control group.

ER stress has been related to the increased expression of cell surface death receptor Fas through the pathway involved with the activation of JNK [39]. In this study we showed that the expression of death receptor Fas (*p* < 0.001) exhibited a large increase with time upon treatment of HepG2 and SMMC-7721 cells up to 24 h with 100 μg/mL **612** (Figure 6A). When SMMC-7721 and HepG2 cells were pre-treated with 100 μg/mL **612** for 24 h, immunofluorescence analysis indicated an increased cell surface distribution of Fas (Figure 6B). Cell treated with **612** (100 μg/mL) followed by 20 ng/mL Fas activating antibody CH-11 for 24 h showed increased apoptosis, as seen in Figure 6C, suggesting that **612** also can serve as an anti-cancer adjuvant to increase the susceptibility of cancer cells to ligand-receptor interaction mediated apoptosis.

## 4. Discussion

Aberrant glycosylation is widely associated with cancer progression [1,40,41]. Hepatocellular carcinoma cells are enriched in glycoproteins carrying *N*- and *O*-glycans, and alterations in glycosylation have been shown to regulate cell-cell interactions [42,43]. Inhibition of glycosylation has been reported to suppress cancer cell metastasis [30,31] and to induce apoptosis in multiple cancer types [28,32,33], highlighting glycosylation pathways as potential anti-cancer targets.

In previous studies, we developed and characterized 2-naphthyl 2-butanamido-2-deoxy-1-thio-β-D-glucopyranoside (**612**) as an inhibitor of β4GalT1 [35,36]. The β4GalT family comprises seven closely related enzymes that share conserved sequence and structural features. Importantly, the reported specificity of **612** refers to inhibition of β4GalT1 and does not inhibit related galactosyltransferases such as β3GalT5 [35]. Nevertheless, given the conserved catalytic architecture among β4GalT family members, interruption of β4GalT1 activity may influence broader β4GalT-associated glycosylation at the cellular level. Therefore, the cellular phenotypes observed in this study are best interpreted in the context of β4GalT-associated glycosylation dependency rather than strict enzyme isoform specificity. The β4GalT family is responsible for the synthesis of *N*-acetyllactosamine chains on *N*- and *O*-glycans of glycoproteins and glycolipids, which serve as precursors for Lewis-related structures. Disruption of these glycan motifs can lead to phenotypic and functional alterations in cancer cells [44]. Moreover, overexpression of β4GalTs has been associated with multidrug resistance in several cancer types [17,18,19], suggesting that interference with β4GalT-associated glycosylation may influence cellular stress responses and therapeutic sensitivity.

In the present study, we primarily focused on HCC although β4GalT-associated glycosylation alterations have been reported in multiple cancer types [12,13,14]. We investigated the cytotoxic and anti-cancer activity of **612** across a panel of cancer cell lines reported to express β4GalT family members (The Human Protein Atlas and [45]). HCC cell lines exhibited the highest cytotoxicity to **612**, whereas non-cancer cell lines (HUVEC and HEK293A) were comparatively resistant (Figure 1 and Table S2). The pronounced sensitivity of HCC cells observed here may reflect the high glycosylation activity and metabolic demands characteristic of hepatocytes, as well as the frequent upregulation of β4GalT family members in liver tumors [15,16]. Whether β4GalT-driven glycosylation represents a broader therapeutic vulnerability across cancers will require systematic evaluation in additional tumor contexts. Nonetheless, our findings establish HCC as a relevant model for interrogating glycosylation-dependent cytotoxic mechanisms. Notably, sensitivity to **612** correlated more closely with elevated β4GalT family activity than with β4GalT1 expression alone, as HepG2 cells displayed relatively low β4GalT1 protein levels yet remain highly sensitive to **612** treatments. Docking analyses further support potential interactions of **612** with conserved residues within the catalytic pocket of β4GalTs, although direct biochemical inhibition beyond β4GalT1 remains to be established.

While compound **612** exhibited selective cytotoxicity in HCC cells, several limitations warrant consideration, including the potential for acquired resistance, effects on glycosylation in non-malignant cells, and off-target activities inherent to glycosylation-modulating compounds. Unlike broad-spectrum glycosylation inhibitors such as tunicamycin, which globally disrupt N-glycosylation and induce profound cellular toxicity, **612** preferentially interferes with β4GalT-associated glycosylation processes. Given these fundamental differences in molecular targets and cellular consequences, we did not directly compare **612** with tunicamycin in this study. Future studies will be required to assess long-term cellular adaptation, toxicity, and translational feasibility of **612** in more physiologically relevant models.

During disease progression, tumor cells can detach from the primary neoplasm, migrate to secondary sites, survive in circulation, extravasate to distinct tissues and adapt to the foreign microenvironments [46]. Adhesion of cancer cells to endothelial cells is a critical step in crossing the endothelial barrier [47]. Treatment of cancer cells with **612** resulted in detectable alterations in cell surface glycosylation, which were accompanied by reduced wound closure and decreased migratory and invasive capacity in vitro (Figure 2). However, these effects may be partially influenced by reduced cell viability and cellular stress responses induced by **612**.

Selectins expressed on activated endothelium are critical receptors mediating cancer cell adhesion and facilitating metastasis [47]. SLe^x^ is a common selectin ligand, has been reported to correlate with metastatic potential in several cancer types, including breast cancer [48]. SLe^x^ is biosynthesis depends on Galβ1-4GlcNAc structures generated by members of the β4GalT family [49]. Consistent with interference of β4GalT-associated glycosylation, we observed downregulation of SLe^x^ expression on the surface of HCC cells following **612** treatments, along with reduced attachment of cancer cells to P- and E-selectins (Figure 3). It should be noted that lectins and glycan-directed antibodies provide semi-quantitative information and may exhibit partial cross-reactivity. Accordingly, alterations in lectin binding and immunofluorescence signals were interpreted as reflecting relative changes in glycosylation patterns rather than definitive identification or absolute quantification of specific glycan structures. These glycosylation changes, together with functional adhesion assays, support an association between β4GalT-associated glycosylation perturbation and impaired cellular motility.

Accordingly, the reduced migration and invasion observed in the Matrigel-coated transwell assays are consistent with impaired cellular motility under conditions of β4GalT1-associated glycosylation interference, although contributions from reduced viability and stress responses cannot be fully excluded. Similar observations have been reported for β4GalT1 inhibitors that attenuate selectin-mediated cancer cell adhesion [25]. As **612** has also been reported to affect other human glycosyltransferases [50], additional glycosylation-dependent processes may be affected by this compound. Therefore, **612** may serve as a useful chemical tool for modulating glycosylation-dependent cellular behaviors in cancers with elevated β4GalT activity, while its potential impact on metastasis will require further investigation in appropriate in vivo models.

The cell cycle is a general phenomenon of eukaryotic cell proliferation, which includes key checkpoints in cell cycle progression such as the G0/G1, S-phase, and G2/M checkpoints. The results presented here show that treatment with **612** induces G2/M phase cell cycle arrest in SMMC-7721 and HepG2 cells (Figure 4). This effect could be due to the induction of ER stress as it was associated with cell cycle arrest at G2/M phase [51].

In addition, both ER stress and Golgi stress contributed to an induction of apoptosis [52,53]. The results indicated that upon the treatment of **612**, the expression of GRP78 increased to regulate protein misfolding. When ER stress is sustained, the unfolded protein response becomes insufficient to restore proteostasis, accompanied by time-dependent upregulation of IRE1α, ATF6, and PERK, which are associated with pro-apoptotic signaling. Apoptosis is a programmed cell death involved in normal physiological and pathological events. The current study shows that **612** exhibits an apoptotic effect against HCC cell cultures. Apoptosis usually occurs via two main pathways. The death receptor-mediated apoptosis pathway, also known as the extrinsic pathway, which is mediated by the interaction of apoptosis inducing ligand with cell surface death receptors, followed by triggering of the receptor-ligand complex formation and caspase-8 activation [54]. The **612** treated cells showed increased expression of Fas and this could be due to the activation of a signaling pathway involving calcium/calmodulin-dependent protein kinase IIgamma (CaMKIIgamma) activation and phosphorylation of JNK [39]. Therefore, Fas activating antibody indicated that extrinsic apoptosis in **612** pre-treated HCC was enhanced (Figure 6).

**Figure 7:**
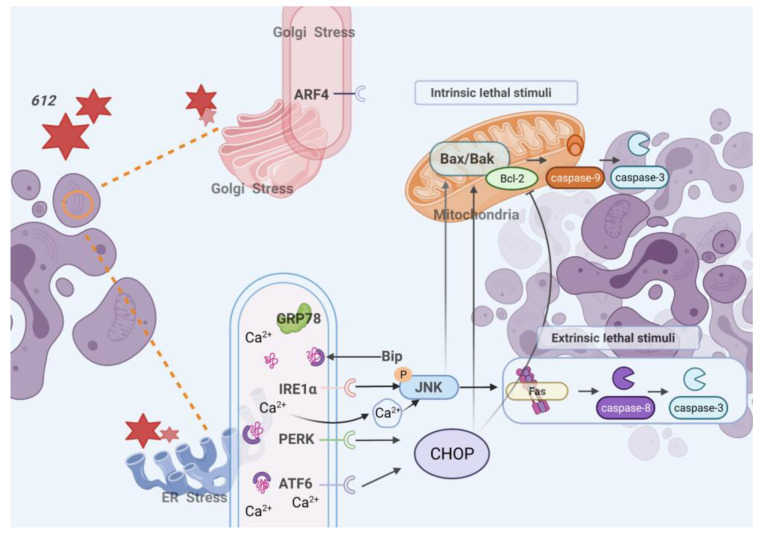
Proposed mechanism underlying the anti-hepatic effects of **612**. Cells treated with **612** underwent ER- and Golgi-stress with the increased expression of signature receptors IRE1α, ATF6, PERK and ARF4 as well as the induction of the downstream signaling pathways. ER-stress led to increased phosphorylation of c-Jun N-terminal kinase (JNK) and expression of a gene regulator CHOP which substantially up-regulated the expression of pro-apoptotic protein Bak/Bax and down-regulated the anti-apoptotic protein Bcl-2 and led to the induction of apoptosis through the intrinsic pathway. Moreover, ER stress can cause the imbalance of calcium homeostasis in the endoplasmic reticulum, which activated P-JNK and up-regulated the expression of Fas death receptor, increased the sensitivity of HCC cells to Fas activating antibody induced extrinsic apoptosis.

The mitochondrial-mediated apoptosis pathway, also known as the intrinsic pathway, can be stimulated by chemotherapeutics [54]. The intrinsic pathway is regulated by Bcl-2 family proteins which control the release of cytochrome c. The pro-apoptotic Bcl-2 family members (such as Bax) promote cytochrome c release, whereas the anti-apoptotic members (such as Bcl-2) inhibit the release of cytochrome c, and have also been implicated in cell-cycle control [55]. ER stress also utilizes the intrinsic pathway [54], and increased expression of ATF6 and IRE1α promotes upregulation of the apoptotic gene regulator protein CHOP. CHOP up-regulated the expression of Bax/Bak, which caused mitochondrial dysfunction and consequently led to the release of cytochrome c. In the current study, the apoptosis rates of HCC cells after treatment with **612** were increased in a time-dependent manner. WB analysis (Figure 5B,C) showed that the expression levels of pro-apoptotic protein Bax, Bak, initiator and effector caspases (caspase-9, caspase-8 and caspase-3) were elevated in cells treated with **612** in a time-dependent manner. Meanwhile, the expression of anti-apoptotic protein Bcl-2 was downregulated by **612**, demonstrating the occurrence of cellular apoptosis through the intrinsic pathway. It has been shown that cell cycle checkpoints appear to link to the intrinsic apoptosis pathway as the protein involved in apoptosis is also essential in cell cycle checkpoints [52,53]. The proposed mechanism of **612** anti-cancer effect was summarized in Figure 7. Inhibitor **612** showed the effects on both the cell cycle arrest and apoptosis, suggesting that **612** exhibited promising anti-cancer effects.

Although compound **612** exhibited clear cytotoxic and mechanistic effects in vitro, several pharmacological considerations warrant discussion. The effective concentrations required in cell-based assays are relatively high, reflecting the physicochemical characteristics of carbohydrate-based glycosyltransferase inhibitors [37], including limited membrane permeability and intracellular availability. These in vitro concentrations therefore do not directly imply clinically achievable exposure levels.

In conclusion, this study demonstrates that the β4GalT1 inhibitor **612** exerts selective cytotoxic effects in HCC with elevated β4GalT-associated glycosylation activity. Treatment with **612** induces ER and Golgi stress, G2/M cell cycle arrest, and activation of both intrinsic and extrinsic apoptotic pathways, accompanied by alterations in cell-surface glycosylation and impaired cellular motility in vitro. These findings highlight the importance of β4GalT1-associated glycosylation in HCC cell survival and stress adaptation and provide mechanistic insight into how interference of glycosylation contributes to cytotoxic anti-tumor phenotypes, although the present work is limited to in vitro analyses. Given the higher concentrations required for cellular efficacy, future studies will need to evaluate pharmacological feasibility, including effects on glycosylation in non-malignant tissues, compound-related toxicity, and strategies to improve potency, formulation, and delivery. Such optimization will be essential to enable meaningful in vivo assessment and to clarify the translational potential of **612**.

## Data Availability

Spatial transcriptomics datasets GSM6177612 used in this study were obtained from GEO datasets (https://www.ncbi.nlm.nih.gov/gds/) (accessed on 25 June 2025). The expression level and clinical data in HCC were extracted from the UCSC Toil RNA-seq recompute database (https://xenabrowser.net/datapages/) (accessed on 11 July 2025).

## Supplementary Materials

The following supporting information can be downloaded at: https://www.mdpi.com/article/10.3390/cells15030251/s1, Figure S1: Sequence alignment of human B4GALT1-7 proteins. Figure S2: Effect of **612** at a concentration of 12.5 µg/mL on HepG2 cell migration. Table S1: Primers used for quantitative real-time PCR analysis of GalTs. Table S2: The IC_50_ values of **612** against various cell lines (*n* = 3, mean ± SD).

## Abbreviations

The following abbreviations are used in this manuscript:

ALT	Alanine aminotransferase
B4GALT	β1,4-galactosyltransferase
BCA	Bicinchoninic acid
BSA	Bovine serum albumin
ConA	Concanavalin A
DAPI	4′,6-diamidino-2-phenylindole
DMSO	Dimethyl sulfoxide
DSL	Datura stramonium lectin
ECL	Enhanced chemiluminescence
ER	Endoplasmic reticulum
FBS	Fetal bovine serum
GTs	Glycosyltransferases
HCC	Hepatocellular carcinoma
HEK293A	Human embryonic kidney cells
HUVEC	Human umbilical vein endothelial cells
IC_50_	Half-maximal inhibitory concentration
Lea	Lewis a
Leb	Lewis b
Ley	Lewis y
MAL	Maackia amurensis lectin
OD	Optical density
PBS	Phosphate-buffered saline
PHA-L	Phaseolus vulgaris leucoagglutinin
PI	Propidium iodide
PVDF	Polyvinylidene fluoride
RCA I	Ricinus communis agglutinin I
ROS	Reactive oxygen species
SD	Standard deviation
SLe^x^	Sialyl-Lewis x
SNA	Sambucus nigra lectin
TBST	Tris-buffered saline with Tween-20
UPR	Unfolded protein response
WB	Western blot
WGA	Wheat germ agglutinin
**612**	2-naphthyl 2-butanamido-2-deoxy-1-thio-β-D-glucopyranoside
