# Supplementary material for: Cytotoxic Effect of a β1,4-Galactosyltransferase Inhibitor in Hepatic Carcinoma Cells
Source: Cells. 2026 Jan 28;15(3):251. doi: 10.3390/cells15030251 (PMC12897170; doi:10.3390/cells15030251)
Supplement: Supplementary file 1 [file cells-15-00251-s001.zip › cells-4075565-supplementary.pdf]

# Supplementary materials

BAD AVG GOOD

```
B4GALT1     MRLREP------LLSGSAAMPGASLQRACRLLVAVCA--LHLGVTLVYY--LAG-RDLSRLPQLVGVSTPLQ
B4GALT2     MSRL----------------LGGTLERVCKAVLLLCL--LHFLVAVILYFDVYA-QHLA-------------
B4GALT3     MLR-------------------RLLERPCTLALLVGS-----QLAVMMYLSLGGFRSLS-------------
B4GALT4     MGFN----------------LTFHLSYKFRLLLLTLCLTVVGWATSNYFVGAI-QEIP-------------
B4GALT5     MRAR----------------R-G-LLRLPRRSLLAALFFFSLS-SSLLYFVYVA-PGIV-------------
B4GALT6     MSVL----------------R-R-MMRVSNRSLLAFIFFFSLS-SSCLYFIYVA-PGIA-------------
B4GALT7     MFPSRRKAAQLPWEDGRSGLLSGGLPRKCSVFHLFVAC-LSLGFFSLLWLQLSC-SG---------------

cons        *                       :                       :


B4GALT1     GGSNSAAAIGQSSGEL-----R---------TG---GARPPPPLGASSQPRPGGDSSPVVDSGPGPASNLTS
B4GALT2     ---FFSRF--SARGPA-----H---------ALHP-AASSSSSSSNCSRPNATASS-----------SGLPE
B4GALT3     ------AL----------------------------FGRDQGPTFDYSHPRDVY-------------SNLSH
B4GALT4     ---KAKEFMANFHKTL------------------I-LGKGKT----------------------------L
B4GALT5     ---NTYLFMMQAQGILIRDNVRTIGAQVYEQVLRSAYAKRNSSVNDSDYPLDLNHS-----------ETFLQ
B4GALT6     ---NTYLFMVQARGIMLRENVKTIGHMI------RLYTNKNSTLNGTDYPEGNNSS-----------DYLVQ
B4GALT7     ----DVARAVRGQG----------------------------------------------------------

cons


B4GALT1     VP--VPH-TTA-LSLPACPEESPLLVGPMLIEFNMPV--DLELVAKQNPNVKMGGRYAPRDCVSPHKVAIII
B4GALT2     VPSALPG-PTA-PTLPPCPDSPPGLVGRLLIEFTSPM--PLERVQRENPGVLMGGRYTPPDCTPAQTVAVII
B4GALT3     LPGAPGG-PPAPQGLPYCPERSPLLVGPVSVSFSPVP--SLAEIVERNPRVEPGGRYRPAGCEPRSRTAIIV
B4GALT4     TN--EAS-TKK-VELDNCPSVSPYLRGQSKLIFKPDL--TLEEVQAENPKVS-RGRYRPQECKALQRVAILV
B4GALT5     TTTFLPEDFTY-FANHTCPERLPSMKGPIDINMSEIGMDYIHELFSKDPTIKLGGHWKPSDCMPRWKVAILI
B4GALT6     TTTYLPENFTY-SPYLPCPEKLPYMRGFLNVNVSEVSFDEIHQLFSKDLDIEPGGHWRPKDCKPRWKVAVLI
B4GALT7     ------Q-ETS-GPPRACPPEPP-----------------------------PEHWEEDASWGPHRLAVLV

cons                         **   *                                ::    .      *:::


B4GALT1     PFRNRQEHLKYWLYYLHPVLQRQQLDYGIYVINQAGDTIFNRAKLLNVGFQEALK-DYDYTCFVFSDVDLIP
B4GALT2     PFRHREHHLRYWLHYLHPILRRQRLRYGVYVINQHGEDTFNRAKLLNVGFLEALKEDAAYDCFIFSDVDLVP
B4GALT3     PHRAREHHLRLLLYHLHPFLQRQQLAYGIYVIHQAGNGTFNRAKLLNVGVREALR-DEEWDCLFLHDVDLLP
B4GALT4     PHRNREKHLMYLLEHLHPFLQRQQLDYGIYVIHQAEGKKFNRAKLLNVGYLEALK-EENWDCFIFHDVDLVP
B4GALT5     PFRNRHEHLPVLFRHLLPMLQRQRLQFAFYVVEQVGTQPFNRAMLFNVGFQEAMK-DLDWDCLIFHDVDHIP
B4GALT6     PFRNRHEHLPIFFLHLIPMLQKQRLEFAFYVIEQTGTQPFNRAMLFNVGFKEAMK-DSVWDCVIFHDVDHLP
B4GALT7     PFRERFEELLVFVPHMRRFLSRKKIRHHIYVLNQVDHFRFNRAALINVGFLESSN-ST--DYIAMHDVDLLP
```

```
cons        *.* * ..*   . ::  .* :::: . .**:.*     **** *:***  *: . .     . : *** :*

B4GALT1     MNDHNAYRCFS-QPRHISVAMDKFGFSLPYVQYFGGVSALSKQQFLTINGFPNNYWGWGGEDDDIFNRLVFR
B4GALT2     MDDRNLYRCGD-QPRHFAIAMDKFGFRLPYAGYFGGVSGLSKAQFLRINGFPNEYWGWGGEDDDIFNRISLT
B4GALT3     ENDHNLYVCDPRGPRHVAVAMNKFGYSLPYPQYFGGVSALTPDQYLKMNGFPNEYWGWGGEDDDIATRVRLA
B4GALT4     ENDFNLYKCEE-HPKHLVVGRNSTGYRLRYSGYFGGVTALSREQFFKVNGFSNNYWGWGGEDDDLRLRVELQ
B4GALT5     ESDRNYYGCGQ-MPRHFATKLDKYMYLLPYTEFFGGVSGLTVEQFRKINGFPNAFWGWGGEDDDLWNRVQNA
B4GALT6     ENDRNYYGCGE-MPRHFAAKLDKYMYILPYKEFFGGVSGLTVEQFRKINGFPNAFWGWGGEDDDLWNRVHYA
B4GALT7     LNEELDYGFPEAGPFHVAS--PELHPLYHYKTYVGGILLLSKQHYRLCNGMSNRFWGWGREDDEFYRRIKGA

cons         .:   *      * *.     .      *  :.**:  *:  ::   **:.* :**** ***::  *:

B4GALT1     GMSISRPNAVVGRCRMIRHSRDKKNEPNPQRFDRIAHTKETM---LSDGLNSLTYQVLDVQ-----RYPL-Y
B4GALT2     GMKISRPDIRIGRYRMIKHDRDKHNEPNPQRFTKIQNTKLTM---KRDGIGSVRYQVLEVS-----RQPL-F
B4GALT3     GMKISRPPTSVGHYKMVKHRGDKGNEENPHRFDLLVRTQNSW---TQDGMNSLTYQLLARE-----LGPL-Y
B4GALT4     RMKISRPLPEVGKYTMVFHTRDKGNEVNAERMKLLHQVSRVW---RTDGLSSCSYKLVSVE-----HNPL-Y
B4GALT5     GYSVSRPEGDTGKYKSIPHHHR-GEVQFLGRYALLRKSKERQ---GLDGLNNLNYFA-NIT-----YDAL-Y
B4GALT6     GYNVTRPEGDLGKYKSIPHHHR-GEVQFLGRYKLLRYSKERQ---YIDGLNNLIYRP-KIL-----VDRL-Y
B4GALT7     GLQLFRPSGITTGYKTFRHLHDPAWRKRDQK-RIAAQKQEQFKVDREGGLNTVKYHVASRTALSVGGAPCTV

cons          .: **         . *           :       .        .*:..   *

B4GALT1     TQITVDIGTP---------------------------------------S--------
B4GALT2     TNITVDIGRPPS---------WPP------------------------RG--------
B4GALT3     TNITADIGTDPRGPRAPSGPRYPPGSSQAFRQEMLQRRPPARPGPLSTANHTALRGSH
B4GALT4     INITVDFWFGA-----------------------------------------------
B4GALT5     KNITVNLTPEL-----------AQ------------------------VNEY------
B4GALT6     TNISVNLMPEL-----------AP------------------------IEDY------
B4GALT7     LNIMLDCDKTA-----------TPW------------------------CTFS-----

cons         :*  :
```

**Figure S1.** Sequence alignment of human B4GALT1-7 proteins.

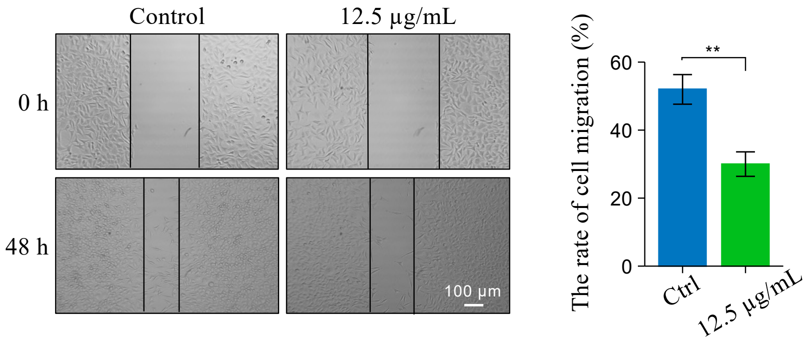


**Figure S2.** Effect of 612 at a concentration of 12.5 µg/mL on HepG2 cell migration.

**Table S1.** Primers used for quantitative real-time PCR analysis of GalTs

| Glycosyltransferases | Forward primer (5'- 3') | Reverse primer (5'- 3') |
|---|---|---|
| B4GALT1 | CTAGCAACTTGACCTCGGT | CATTTGGGTTCTGCTTTGCC |
| B4GALT2 | GCATAACGAACCTAACCCTCAG | GCCCAATGTCCACTGTGATA |
| B4GALT3 | GTAACCTCAGTCACCTGCC | ATTCCGCTCCACAATCTCTG |
| B4GALT4 | ACTTCGTGGGTGCCATTCAAGAGA | AAGGAGACACAGAAGGGCAGTTGT |
| B4GALT5 | TGGAACAGAGTACAGAATGCAG | CCTTGCCGTTCTTTTGACTTC |
| B4GALT6 | CTTCACATACTCACCATACCTCC | CCCCTGGCTCAATATCTAAATCC |
| B4GALT7 | GGTGGACCACTTCAGGTTCA | TGCATGGCAATGTAGTCCGT |

**Table S2.** The $IC_{50}$ values of ***612*** against various cell lines ($n$ = 3, mean ± SD).

| Cell line | | $IC_{50}$ (µg/mL) |
|---|---|---|
| Human umbilical vein endothelial cells | HUVEC | 331.6 ± 8.3 |
| Human embryonic kidney cells | HEK293A | 309.2 ± 4.7 |
| Human breast cancer cells | MDA-MB-231 | 163.1 ± 4.6 |
| Human colorectal adenocarcinoma | SW480 | 149.0 ± 0.9 |
| Human breast cancer cells | MCF-7 | 133.7 ± 1.7 |
| Human lung carcinoma cells | A549 | 99.0 ± 0.8 |
| Human hepatocarcinoma cells | SMMC-7721 | 74.3 ± 0.9 |
| Human hepatocellular carcinoma cells | HepG2 | 59.2 ± 0.7 |
